# Appropriateness and inappropriate medication predictors of stress ulcer prophylaxis in the intensive care unit

**DOI:** 10.3389/fphar.2024.1401335

**Published:** 2025-01-07

**Authors:** Pan Zhang, Siyang Wang, Tingting Zhi, Naobei Ye, Haonan Sun, Xingyu Qin, Shuhan Xu, Ruiqin Zhang

**Affiliations:** ^1^ College of Pharmacy, Shanxi Medical University, Taiyuan, Shanxi, China; ^2^ Department of Pharmacy, Second Hospital of Shanxi Medical University, Taiyuan, Shanxi, China; ^3^ The Second Clinical Medicine School, Shanxi Medical University, Taiyuan, Shanxi, China

**Keywords:** stress ulcer prophylaxis, intensive care unit, rational drug use, proton pump inhibitors, operating characteristic curve

## Abstract

**Introduction:**

Preventive drugs for stress ulcers are widely and unreasonably used in the Intensive Care Unit (ICU). This study aims to examine the appropriate utilization of medications for stress ulcer prophylaxis (SUP) and identify factors that contribute to the inappropriate use of these medications in the ICU of the Second Hospital of Shanxi Medical University.

**Methods:**

Patient cases admitted to the ICU during the period from May 2022 to May 2023 were extracted from the hospital’s information management system. Single-factor analysis and multivariate logistic regression model analysis were performed using the SPSS to identify factors associated with inappropriate medication for prophylaxis. The efficacy of this predictive model was assessed through the use of the Receiver Operating Characteristic Curve (ROC), while the Hosmer test was utilized to evaluate the model fit.

**Results:**

This study included a total of 651 patient cases that met the inclusion criteria. Among these cases, 48.39% were found to have received inappropriate medication of SUP. The analysis revealed a significant association between inappropriate medication and partial transfer to departments (P < 0.05), as well as the use of anticoagulants (P = 0.009) in the prophylaxis group. In the non-prophylaxis group, the multifactorial logistic analysis indicated a significant correlation between inadequate prescriptions and partial transfer to departments (P < 0.05), as well as the presence of artificial airways (P < 0.01).

**Conclusion:**

There is a notable prevalence of inappropriate SUP in the ICU of this hospital. Attention should be paid to the SUP of some patients transferred to the department, the use of anticoagulants and the presence of artificial airway.

## Introduction

Stress ulcers (SU) denotes acute mucosal lesions in the gastrointestinal tract that arise during diverse severe stress circumstances. In instances of heightened severity, it can result in gastrointestinal bleeding, and in some cases, even perforation, thereby exacerbating pre-existing ailments and elevating mortality rates (Bo et al., 2018). Based on systematic review and network meta-analysis, prophylactic use of drugs can reduce gastrointestinal bleeding in high-risk patients (Wang et al., 2020). Prophylaxis medications for stress ulcers are extensively employed in the Intensive Care Unit (ICU), with a significantly higher proportion of physicians prescribing these medications.An observational study targeting ICUs showed that 92.9% of ICU patients received stress ulcer prevention therapy, but the rationality of its was low (Franchitti et al., 2020).

The common drugs used to Stress Ulcer Prophylaxis (SUP) are acid-suppressive medications (ASMs), such as Proton pump inhibitors (PPIs) and histamine-2 receptor antagonists (H2RAs) (Ye et al., 2020). Nevertheless, there is currently no consensus regarding the comparative efficacy and risk of adverse reactions between these two medication classes. A meta-analyses have indicated that there is no statistically significant disparity in the probability of pneumonia occurrence between the two drug classes (Barkun et al., 2012). In comparison to alternative treatments such as H2RAs, there is a dearth of high-quality research studies demonstrating a distinct advantage of PPIs over H2RAs (Barletta and Sclar, 2014; Song et al., 2021). Both PPIs and H2RAS can be used to reduce the risk of clinically important bleeding (Ye et al., 2020). PPIs prevent aspirin-induced gastrointestinal bleeding better in comparison to H2RAs (Szabó et al., 2017). However, compared with H2RAs use, treatment with PPIs for SUP was associated with a 38.6% increased risk of-acquired Clostridioides difficileinfection (Azab et al., 2017).

Nevertheless, the suitability of this prophylactic approach necessitates further examination. Despite the presence of numerous guidelines and consensus statements, both nationally and internationally, pertaining to SUP, the inappropriate utilization of such medications continues to persist as a prevalent concern (Association, 2015; ASHP Therapeutic Guidelines on Stress Ulcer Prophylaxis, 1999; Bo et al., 2018; Ye et al., 2020; National Health Commission of the People 's Republic of China, 2020). A studies concentrating on ICU patients have documented that 82% receive acid-suppressive therapy without appropriate indications (Frandah et al., 2014).

Moreover, it should be noted that not all patients necessitate SUP, as the administration of ASMs can potentially lead to an elevated risk of pneumonia and difficult-to-treat Clostridioides difficileinfection (Barletta and Sclar, 2014). The excessive use of prophylactic measures may result in adverse reactions and concurrently impose a financial burden on patients seeking medical treatment. Consequently, this study primary objective was to evaluate the appropriateness of medication utilization, identify demographic and clinical factors associated with inappropriate medication usage, and Promote the rational use of SUP drugs to some extent.

## Materials and methods

### Study population

A retrospective study was conducted at a tertiary teaching hospital located in the Shanxi province of northern China. The inclusion criteria for this study consisted of patients who were initially admitted to the ICU between 1 May 2022, and 31 May 2023, and were at least 18 years old. On the other hand, the exclusion criteria included patients who 1) had been prescribed ASMs for the treatment of various conditions such as upper gastrointestinal bleeding, MALT lymphoma, gastroesophageal reflux disease (GERD), gastrointestinal ulcers, erosive esophagitis, gastrinoma (Zollinger-Ellison syndrome), eosinophilic esophagitis, Barrett’s esophagus, *Helicobacter pylori* infection, or experienced upper abdominal pain within the month preceding their admission; 2) missing clinical data; 3) ICU length of stay less than 2 days; 4) a history of peptic ulcer or gastrointestinal bleeding within the year before admission.

### Criteria establishment

The criteria for evaluating the appropriateness of SUP medication and risk factors were established based on published evidence-based guidelines from multiple countries, expert consultations, and literature on clinical practices (ASHP Therapeutic Guidelines on Stress Ulcer Prophylaxis, 1999; Bo et al., 2018; Ye et al., 2020; National Health Commission of the People 's Republic of China, 2020). This study aimed to establish the appropriate utilization of SUP in patients who presented with at least one major risk factor or a minimum of two moderate risk factors while being administered PPIs or H2RAs (as outlined in Table 1). The formulation and dosage of the medications were determined in accordance with drug instructions and guidelines.

**TABLE 1:** Risk factors of stress ulcer.

Major risk factors	Moderate risk factors
Mechanical ventilation without enteral nutrition( ≥ 48 h)	Sepsis
Disturbances of blood coagulation	Corticosteroid therapy(>200 mg of hydrocortisone equivalent daily)
Severe traumatic brain injury	ICU stay of >1 week
Severe burn (area >30 or third degree burn area >10%)	Fecal occult blood lasted ≥ 3 days
Severe trauma. multiple trauma (ISS score >16)	
Acute renal failure	
Major surgery	
Multiple organ dysfunction syndrome	
Shock	
Hepatic insufficiency	
Spinal cord injury	

### Data collection

Patient data, including demographic information such as age (in years), gender, weight, current smoking status (yes or no), and alcohol consumption (yes or no), were collected through a systematic search and retrieval process utilizing unique hospital identification numbers assigned to each patient.

The data obtained from medical records encompassed a range of variables, including admission diagnosis, comorbidities (such as chronic obstructive pulmonary disease, liver disease, cancer, etc*.*), admission and discharge dates, transfers to different departments, surgical procedures, duration of surgery, surgical risk level, pertinent laboratory data, medication details, dietary information, enteral nutrition status (yes or no), mechanical ventilation status (yes or no), gastric fluid color, stool color, as well as medication-related adverse reactions during hospitalization.Acute Physiology and Chronic Health Evaluation (APACHEⅡ) score = Acute Physiology score + Age score + Chronic Health score, which is used to assess the health status of patients.

### Statistical analysis

A comprehensive analysis was undertaken to evaluate the suitability of SUP and determine the factors linked to inappropriate use of SUP medication. Patients were categorized into two groups based on their utilization of SUP medications: the prophylaxis group and the non-prophylaxis group. The appropriateness of SUP measures was determined by the presence of either one major risk factor or two or more minor risk factors. In the prophylaxis group, patients were divided into two subgroups: a appropriate subgroup, which had indications present with appropriate formulation and administration method, and an inappropriate subgroup, which either lacked indications or had inappropriate formulation, dosage, or administration method. In the non-prophylaxis group, patients were categorized into a appropriate subgroup, which lacked indications and medication, and an inappropriate subgroup, which had indications present but lacked medication.

Within the prophylaxis group, comparisons were conducted between these two subgroups to ascertain predictive factors linked to inappropriate medication. Likewise, comparisons were performed between the two subgroups within the non-prophylaxis group to identify predictive factors associated with the absence of medication (insufficient prescription).

Mean ± standard deviation were employed to represent population demographics and clinical data that adhered to a normal distribution. 95%CI is used to represent the accuracy and confidence of the sample. Non-normally distributed values were represented using the median and quartiles.The data analysis initially involved the use of single-factor logistic regression. In cases where clinical variables exhibited statistically significant differences in the single-factor analysis, a multiple-factor logistic regression model was employed. Subsequently, a predictive model for inappropriate medication was developed and constructed based on the obtained results. The efficacy of this predictive model was assessed through the use of the Receiver Operating Characteristic Curve (ROC), while the Hosmer test was utilized to evaluate the model fit. All statistical analyses were performed using the SPSS V19.0 software package. Two-tailed tests were conducted at a significance level of *p* < 0.05 to determine statistical significance. The threshold for determining statistical significance was set at 0.05 (*p* < 0.05).

## Results

### Characteristics of the study population

Initially, a total of 1,108 patients were included in the study based on the predetermined inclusion criteria. Subsequently, 457 patients were excluded after applying the exclusion criteria, resulting in a final sample size of 651 patients. Among these patients, 58.83% received ASMs for SUP, with 39.69% classified as appropriate users. Furthermore, the majority of the participants were male, accounting for 63.19% of the total sample. Among the 268 patients who did not receive ASMs prescriptions for SUP, 68.66% were classified as appropriate users. Among the patients included in the study, a total of 664 instances of major and minor risk factors were identified. The most prevalent major risk factor was patients on mechanical ventilation without enteral nutrition, accounting for 31.02%, followed by patients undergoing complex surgeries with severe difficulty, accounting for 21.84%.

**FIGURE 1:**
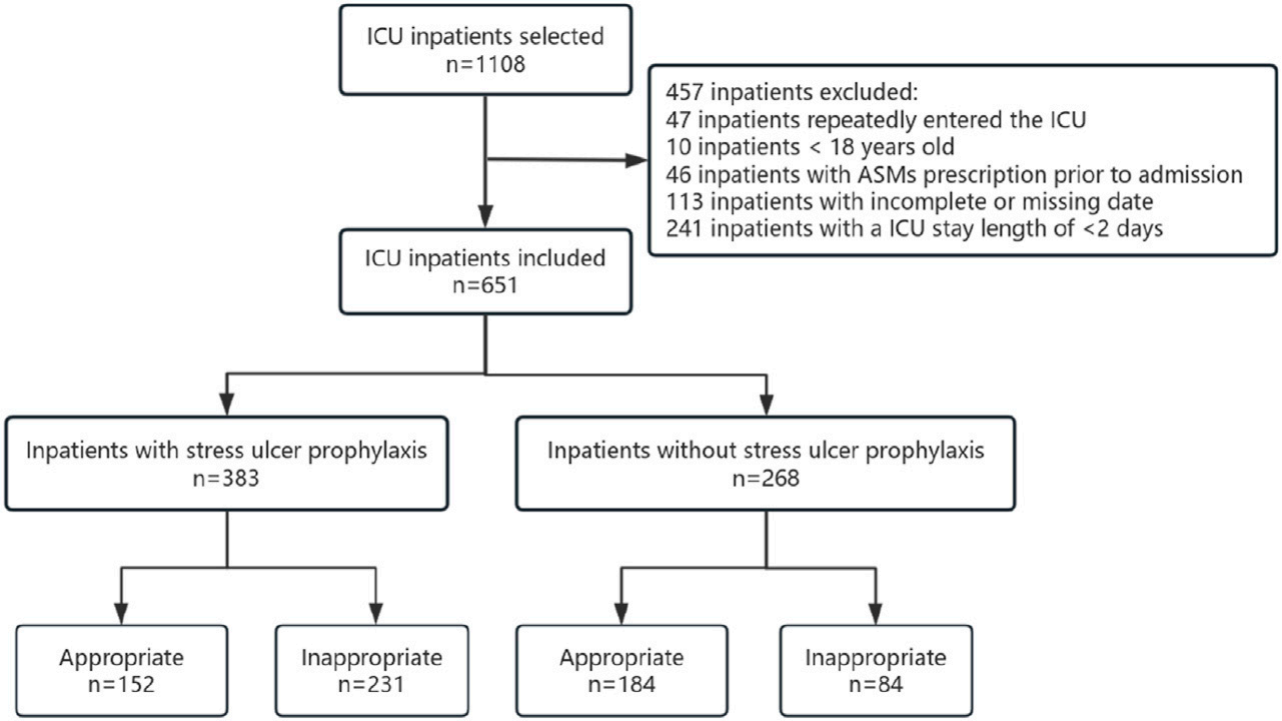
Screening and grouping of ICU patients.

A total of 664 instances of major and minor risk factors were identified among the included patients. The most prevalent major risk factor was patients on mechanical ventilation without enteral nutrition, accounting for 31.02%. Among the minor risk factors, a significant number of cases (188) were attributed to patients with an ICU length of stay exceeding 1 week, constituting 28.31% of the occurrences (Figure 1; Table 2).

**TABLE 2:** Demographic information of prophylaxis group and non-prophylaxis group of ICU patients.

Date	Prophylaxis group		Non-prophylaxis group	
Gender (%)				
Male	242	(63.19)	143	(53.36)
Female	141	(36.81)	125	(46.64)
Age, year (%)				
18-40	68	(17.75)	49	(18.28)
41-65	151	(39.43)	92	(34.33)
>65	164	(42.82)	127	(47.39)
Cost (Q1, Q3)				
Drugs	7492.61	(3350.33,16207.30)	3164.76	(1303.74,6916.74)
Total	27488.32	(14612.82,55788.23)	15408.82	(9354.97,26061.75)
Major risk factors (%)				
Mechanical ventilation without enteral nutrition (≥48 h)	148	(22.36)	58	(8.76)
Major surgery	114	(17.22)	31	(4.68)
Disturbances of blood coagulation	49	(7.40)	1	(0.15)
Severe trauma, multiple trauma (ISS score >16)	18	(2.72)	1	(0.15)
Spinal cord injury	18	(2.72)	1	(0.15)
Shock	10	(1.51)	4	(0.60)
Severe traumatic brain injury	8	(1.21)	0	(0.00)
Hepatic insufficiency	1	(0.15)	0	(0.00)
Total	358	(54.08)	96	(14.50)
Moderate risk factors (%)				
ICU stay of >1 week	134	(20.24)	54	(8.16)
Sepsis	10	(1.51)	1	(0.15)
Fecal occult blood lasted ≥3 days	1	(0.15)	0	(0.00)
Total	145	(21.90)	55	(8.31)

Cost(Q1,Q3):Cost(Quartile1,Quartile3).

### ASM prescription data for prophylaxis group patients

In the prevention group, a total of 383 patients received ASMs. The majority of these patients were prescribed PPIs. Specifically, 183 patients were prescribed lansoprazole, accounting for 41.22% of the total. The least commonly used PPI was pantoprazole, which was prescribed for only one patient, accounting for 0.23%. Additionally, 61 patients in this group were prescribed a combination of two ASMs. The total duration of medication for the appropriate group was found to be 1785 days, with a median duration of 3 days. The range of durations varied from a minimum of 1 day to a maximum of 70 days. Conversely, the inappropriate group had a total medication duration of 765 days, with a median duration of 5 days. The range of durations for this group ranged from 1 day to 32 days. In terms of administration method, a significant majority of patients (77.93%) received intravenous administration (Table 3).

**TABLE 3:** ASMs usage in prophylaxis group.

		Appropriate		Unappropriate		Total	
Routes of administration (%)	Intravenous	152	(34.23)	194	(43.69)	346	(77.93)
	Oral	9	(2.03)	89	(20.05)	98	(22.07)
Frequency of administration (%)	Bid	110	(24.77)	151	(34.01)	261	(58.78)
	Tid	2	(0.45)	5	(1.13)	7	(1.58)
	Qd	49	(11.04)	127	(28.60)	176	(39.64)
Kinds of medicine (%)	Lansoprazole	66	(14.86)	117	(26.35)	183	(41.22)
	Omeprazole	29	(6.53)	45	(10.14)	74	(16.67)
	Esomeprazole	24	(5.41)	89	(20.05)	113	(25.45)
	Pantoprazole	0	(0.00)	1	(0.23)	1	(0.23)
	Rabeprazole	0	(0.00)	3	(0.68)	3	(0.68)
	Cimetidine	42	(9.46)	28	(6.31)	70	(15.77)
Duration of administration (days) (Q1,Q3)		3	(2,6)	5	(3,9)	4	(2,8)

Bid:bis in die; Tid:ter in die; Qd:quaque die.Duration of administration(days) (Q1,Q3): Duration of administration(days) (Quartile1,Quartile3).

### Evaluation of inappropriate use of SUP

We conducted an analysis of the daily medication usage for patients and summarized the reasons for inappropriate SUP prescription (Table 4).

**TABLE 4:** Statistics of SUP inappropriate results.

Results		Prophylaxis group		Non-prophylaxis group	
Non-indicative drug use		136	(53.54)	0	(0.00)
Overdose use		46	(18.11)	0	(0.00)
Unreasonable dosage form		12	(4.72)	0	(0.00)
Not timely medication	Shock	3	(1.18)	3	(3.53)
	Major surgery	14	(5.51)	31	(36.47)
	Disturbances of blood coagulation	15	(5.91)	1	(1.18)
	Mechanical ventilation without enteral nutrition(≥48h)	21	(8.27)	47	(55.29)
	Severe trauma, multiple trauma (ISS score >16)	2	(0.79)	1	(1.18)
	Spinal cord injury	4	(1.57)	1	(1.18)
	Severe traumatic brain injury	1	(0.39)	1	(1.18)
Total		254	(100.00)	85	(100.00)

### Analysis of factors related to SUP inappropriate prescription

We conducted a statistical analysis of the demographic and clinical characteristics between the appropriate and inappropriate groups in the prophylaxis group, as shown in Supplementary Table S1. For the demographic and clinical characteristics of the non-prophylaxis group, please refer to Supplementary Table S2.

The findings from the single-factor analysis of patients receiving medication indicated a significant association between inappropriate prescription and factors such as the department of admission, the presence of artificial airways, and the utilization of anticoagulants and glucocorticoids. The findings from a logistic regression analysis involving multiple factors revealed a statistically significant association between inappropriate prescription and the department of admission (specifically, hepatobiliary surgery, infectious disease ward, vascular surgery, gastroenterology, thyroid department, and cardiology) among patients receiving medication. Additionally, the use of anticoagulants was also found to be significantly correlated with inappropriate prescription (Supplementary Table S3).

Furthermore, a single-factor analysis conducted on patients who did not receive medication demonstrated a significant correlation between under-prescription and the department of admission, liver disease, tumor presence, the use of artificial airways, and APACHE II score.The findings from the logistic regression analysis demonstrate a significant association between the department of admission (specifically, orthopedics,hepatobiliary surgery, cardiothoracic surgery, infectious disease ward, cardiology) and the presence of artificial airways with under-prescription in patients who were not administered medication (as shown in Supplementary Table S4).

### Prediction model and efficacy assessment of factors related to SUP inappropriate prescription

**FIGURE 2:**
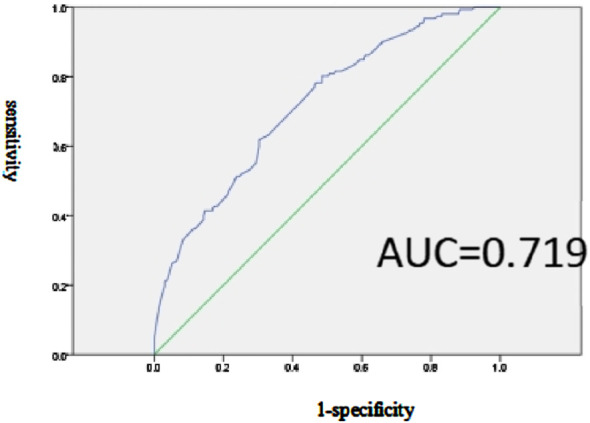
Effectiveness evaluation of the prediction model of SUP inappropriate prescription related factors in the prevention group.

The multiple-factor logistic regression analysis further indicates a predictive model for inappropriate SUP in the prophylaxis group, with the equation Logit(P) = −1.922 + 2.851 × Hepatobiliary surgery +1.473 × Infectious disease +2.259 × Vascular surgery +2.214 × Gastro colorectal ward +1.778 × Cardiology department +2.242 × Other departments - 0.711 × Combined use of one anticoagulant, where P represents the probability of adverse drug reaction occurrence. Additionally, the ROC curve analysis reveals an area under the curve (AUC) of 0.719 (95% CI: 0.667–0.770, *p* < 0.001).The sensitivity and specificity of this model, with a cutoff point set at *p* = 0.318, are 80.3% and 51.5%, respectively, as shown in Figure 2.

**FIGURE 3:**
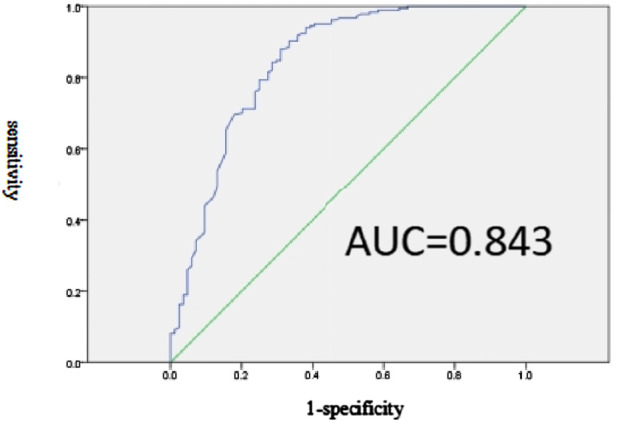
Effectiveness evaluation of the prediction model of SUP inappropriate prescription related factors in non-prevention group.

The predictive model for inappropriate SUP in the non-prophylaxis group is represented by the equation Logit(P) = −3.694 - 1.786 × Orthopedics - 3.211 × Hepatobiliary surgery - 2.86 × Cardiology department - 2.438 × Infectious disease - 3.294 × Use of artificial airway. The sensitivity and specificity of this model, with a cutoff point set at *p* = 0.57, are 88.0% and 69.0%, respectively, as shown in Figure 3.

## Discussion

This study encompassed a cohort of 651 patients, with a notable predominance of male individuals, a trend that can be attributed to the demographic composition. Among the patients receiving prophylaxis, 60.31% had prescription errors, with 53.54% of these errors being attributed to medication without indication. This finding is in line with a SUP study conducted in Lebanon (Zeitoun et al., 2011), where medication without indication accounted for 67%, further supporting our results.In a tertiary hospital in Jordan, 86% of SUP drugs used were unnecessary (Alqudah et al., 2016). Several published investigations have documented overprescription rates ranging from 26.75% to 48%, all of which were lower than the findings of our study. However, it is important to acknowledge that this disparity may be influenced by variations in the criteria used for assessment (Masood et al., 2018; Wijaya et al., 2020).

Upon careful examination, it was determined that 58.83% of patients were administered SUP medication, this rate is lower than the usage rates reported in some studies, which was 85% in ICU patients (Farley et al., 2013). Currently, there exists no consensus regarding the optimal choice of prophylaxis medications. Nonetheless, some studies propose that PPIs may represent the most efficacious option when compared to H2RAs (Bardou et al., 2015; Szabó et al., 2017). Regarding SUP, the utilization rate of PPIs ranges from 96.1% to 100%, surpassing that of H2RAs by a significant margin (Li et al., 2022; Masood et al., 2018). Despite the limited availability of robust evidence, certain studies still advocate for the preference of PPIs over H2RAs (Madsen et al., 2014). The 2018 recommendations in China also endorse PPIs as the primary medication for SUP (Bo et al., 2018). However, some research suggests that the utilization of PPIs may not substantially decrease the incidence of gastrointestinal bleeding and could potentially elevate the risk of hospital-acquired pneumonia (Sun et al., 2019). Furthermore, the prolonged use of PPIs is associated with an augmented likelihood of developing pancreatic cancer (Brusselaers et al., 2020). Furthermore, in comparison to H2RAs, the utilization of PPIs may potentially contribute to an increased mortality rate among patients (Lee et al., 2021). Our study observed that a significant proportion (84.23%) of individuals under medication were prescribed PPIs, which is consistent with the findings of numerous other investigations (Krag et al., 2015; Li et al., 2022; Masoompour et al., 2017). Among the PPIs employed in our study, lansoprazole and esomeprazole were the most frequently administered. Both of these medications undergo metabolism via the enzyme CYP3A4 and CYP2C19 (Fan and Luo, 2023). Lansoprazole had the highest rate of use for SUP in a perioperative study, which is consistent with our results (Xing et al., 2021). Lansoprazole, a derivative of omeprazole, exhibits enhanced bioavailability due to modifications in its side chain. On the other hand, esomeprazole is characterized by a relatively sluggish metabolic process, negligible impact on normal gastric acid secretion, and a reduced first-pass effect, thereby yielding favorable outcomes in terms of ameliorating clinical symptoms for patients.In an international randomized trial, pantoprazole use was associated with a reduction in clinically important bleeding among patients receiving invasive ventilation (Cook et al., 2024).

Moreover, it is crucial to promptly discontinue medication when a patient’s high-risk condition is alleviated in order to minimize the probability of adverse reactions. It is worth noting that the longer a patient remains hospitalized, the higher the susceptibility to iatrogenic infections. When comparing the subgroups within the prevention group, it becomes apparent that the subgroup receiving inappropriate treatment had a prolonged duration of medication. This correlation was also observed in a specific study where the average treatment duration was approximately 5 days, aligning with our own findings (Nasser et al., 2010).

Presently, there is a lack of consensus and clarity regarding the dosage and administration of drugs in the ICU, both domestically and internationally (Ye et al., 2020). Consequently, we amalgamated expert recommendations, medication guidelines, and package inserts, utilizing the maximum therapeutic dose as the upper limit, in order to establish specific drug limitations (Bo et al., 2018; National Health Commission of the People 's Republic of China, 2020). The daily dosage for oral tablets and capsules, such as lansoprazole enteric-coated tablets 30mg, esomeprazole enteric-coated capsules 40mg, omeprazole enteric-coated capsules 40mg, pantoprazole sodium enteric-coated tablets 40mg, and rabeprazole sodium enteric-coated tablets 20mg, was administered once a day. Conversely, injectables, including injectable lansoprazole 30mg/dose, injectable esomeprazole sodium 40mg/dose, injectable omeprazole sodium 40mg/dose, and intravenous cimetidine injection 200mg/dose, were administered twice a day. In a study centered on the utilization of SUP drugs in surgical patients, the rate of injectable drug usage was found to be 86.25%, which is higher than observed in our study (Wijaya et al., 2020).

In accordance with prior scholarly works, the administration of intravenous injections to patients who are capable of taking oral medications is deemed inappropriate (Nourian et al., 2018). Previous studies have reported incidences of inappropriate drug administration at a rate ranging from 42.67% to 66.67% (Luo et al., 2018; Wijaya et al., 2020). It is important to note that prolonged use of PPIs may result in complications such as fractures, hypomagnesemia, Clostridioides difficileinfection, acute kidney injury, and chronic kidney disease (Clarke et al., 2022).

Additionally, the use of anticoagulants was identified as a significant predictor for inappropriate SUP use. This finding aligns with a separate study that also considered the concurrent use of anticoagulants as a predictive factor for SUP prescription errors, supporting the validity of our research findings (Li et al., 2022). However, contrary to our findings, another study identified the use of glucocorticoids as a significant predictive factor for inappropriate SUP (OR = 0.02, 95% CI = 0.01-0.04) (Schepisi et al., 2016).

Presently, a multitude of domestic and international studies have demonstrated that implementing pharmacists’interventions can effectively address inappropriate prescribing, while simultaneously mitigating the economic strain on patientss (Choi et al., 2020; Luo et al., 2018; Masood et al., 2018; Orelio et al., 2021).

Based on the available literature, the prevailing research in our nation primarily concentrates on the improper utilization of PPIs. Consequently, this investigation serves as the initial examination of the determinants that forecast the inappropriate use of SUP medications among ICU patients.The sample size of our study is deemed adequate to reasonably reflect the SUP medication landscape within the ICU to a certain extent.

## Strengths and limitations

We are confident that this study will contribute to a comprehensive comprehension of the prescribing patterns of clinical practitioners and furnish effective strategies for the management of SUP, thereby benefiting researchers and decision-makers alike. However, it is important to acknowledge the limitations of our study. Firstly, the inclusion of only a tertiary A hospital may limit the generalizability of our findings to other regions in terms of the prophylactic medication situation. Secondly, our focus on adult patients in the ICU may restrict the applicability of our results to other departments and pediatric populations. Lastly, the retrospective nature of this study raises concerns about the authenticity and completeness of the data.

## Conclusion

This retrospective study found that 60.31% of these patients may not have actually needed acid-suppressive medications for SUP. Furthermore, the study identifies a significant correlation between the choice of ICU department and the inappropriate use of anticoagulants in the prophylactic group. Additionally, the presence of artificial airways in the non-prophylactic group is associated with inadequate prescriptions. These findings underscore the need for targeted interventions in these specific areas.

We have identified predictive factors linked to inappropriate SUP, thereby offering valuable insights for subsequent clinical pharmacist interventions and medication education initiatives to promote appropriate and efficacious medication utilization.

## Data Availability

The data analyzed in this study is subject to the following licenses/restrictions: All data were from the ICU of the same hospital and were collected retrospectively. Requests to access these datasets should be directed to Zhang Pan, quanliyifu1016@163.com.
