# Supplementary material for: Appropriateness and inappropriate medication predictors of stress ulcer prophylaxis in the intensive care unit
Source: Front Pharmacol. 2025 Jan 7;15:1401335. doi: 10.3389/fphar.2024.1401335 (PMC11746069; doi:10.3389/fphar.2024.1401335)
Supplement: Supplementary file 1 [file Table1.pdf]

STable 1 Demographic and clinical characteristics statistics for the prophylaxis group

Characteristics	Total(n=383)		Appropriate（n=152）		Unappropriate（n=231）	
Gender						
Male	242	(63.19)	87	(57.24）	155	(67.10)
Female	141	(36.81)	65	(42.76）	76	(32.90)
Age（years）						
Median(Q1,Q3)	62	(49,74)	60	(52,71)	65	(48,75)
18-40	68	(17.75)	24	(15.79）	44	(19.05)
41-65	151	(39.43)	74	(48.68）	77	(33.33)
≥66	164	(42.82)	54	(35.53）	110	(47.62)
Transferred to department						
ICU	29	(7.57)	3	(1.97）	26	(11.26)
Orthopedics	110	(28.72)	39	(25.66）	71	(30.74)
Hepatobiliary surgery	56	(14.62)	32	(21.05）	24	(10.39)
Military sursery	23	(6.01)	14	(9.21）	9	(3.90)
Cardiothoracic surgery	10	(2.61)	3	(1.97）	7	(3.03)
Obstetrical department	13	(3.39)	4	(2.63）	9	(3.90)
Infectious disease	7	(1.83)	2	(1.32）	5	(2.16)
Vascular surgery	12	(3.13)	6	(3.95）	6	(2.60)
Gastro colorectal ward	25	(6.53)	14	(9.21）	11	(4.76)
Geriatrics	11	(2.87)	0	(0.00）	11	(4.76)
Gynaecology	18	(4.69)	13	(8.55）	5	(2.16)
Thyroid department	14	(3.66)	5	(3.29）	9	(3.90)
Cardiology department	6	(1.57)	2	(1.32）	4	(1.73)
Others	49	(12.79)	15	(9.87）	34	(14.72)
Diabetes						
No	323	(84.33)	135	(88.82）	188	(81.39)
Yes	60	(15.67)	17	(11.18）	43	(18.61)
Chronic obstructive pulmonary disease						
No	379	(98.96)	151	(99.34）	228	(98.70)
Yes	4	(1.04)	1	(0.66）	3	(1.30)
Hypertension						
No	278	(72.58)	105	(69.08）	173	(74.89)
Yes	105	(27.42)	47	(30.92）	58	(25.11)
Hepatopathy						
No	313	(81.72)	125	(82.24）	188	(81.39)
Yes	70	(18.28)	27	(17.76）	43	(18.61)
Tumor						
No	315	(82.25)	118	(77.63）	197	(85.28)
Yes	68	(17.75)	34	(22.37）	34	(14.72)
Artificial airway						
No	240	(62.66)	84	(55.26）	156	(67.53)
Yes	143	(37.34)	68	(44.74）	75	(32.47)
APACHE Ⅱ score						

Median(Q1,Q3)	25 (19,34)	26 (20,34)	25 (18,35)
Method of payment			
Basic medical insurance for urban employees	62 (16.19)	20 (13.16)	42 (18.18)
Basic medical insurance for urban and rural residents	97 (25.33)	42 (27.63)	55 (23.81)
Full public expense	5 (1.31)	0 (0.00)	5 (2.16)
Fullscale motor test sef cost	22 (5.74)	10 (6.58)	12 (5.19)
Other social insurance	2 (0.52)	1 (0.66)	1 (0.43)
Others	195 (50.91)	79 (51.97)	116 (50.22)
Allergic history			
No	362 (94.52)	140 (92.11)	222 (96.10)
Yes	21 (5.48)	12 (7.89)	9 (3.90)
Smoking history			
No	297 (77.55)	118 (77.63)	179 (77.49)
Yes	6 (1.57)	34 (22.37)	52 (22.51)
Drinking history			
No	327 (85.38)	131 (86.18)	196 (84.85)
Yes	56 (14.62)	21 (13.82)	35 (15.15)
Number of NSAIDs			
0	371 (96.87)	150 (98.68)	221 (95.67)
1	12 (3.13)	2 (1.32)	10 (4.33)
Number of anticoagulants			
0	269 (70.23)	122 (80.26)	147 (63.64)
1	114 (29.77)	30 (19.74)	84 (36.36)
Number of glucocorticoid			
0	94 (24.54)	39 (25.66)	55 (23.81)
1	244 (63.71)	104 (68.42)	140 (60.61)
2	40 (10.44)	8 (5.26)	32 (13.85)
3	5 (1.31)	1 (0.66)	4 (1.73)

The data are presented as numbers (proportions) or the median (interquartile range).Median(Q1,Q3):Median(Quartile1,Quartile3).

STable 2 Statistics of population and clinical characteristics of non-prevention group

Characteristics	Total(n=268)	Appropriate（n=184）	Unappropriate（n=84）
Gender			
Male	143 (53.36)	93 (50.54)	50 (59.52)
Female	125 (46.64)	91 (49.46)	34 (40.48)
Age（years）			
Median(Q1,Q3)	64 (48,78)	67 (49,81)	62 (47,73)
18-40	49 (18.28)	33 (17.93)	16 (19.05)
41-65	92 (34.33)	57 (30.98)	35 (41.67)
⩾66	127 (47.39)	94 (51.09)	33 (39.29)
Transferred to department			
ICU	34 (12.69)	29 (15.76)	5 (5.95)
Orthopedics	120 (44.78)	94 (51.09)	26 (30.95)
Hepatobiliary surgery	15 (5.60)	4 (2.17)	11 (13.10)

Military sursery	9	(3.36)	5	(2.72)	4	(4.76)
Cardiothoracic surgery	4	(1.49)	2	(1.09)	2	(2.38)
Obstetrical department	18	(6.72)	10	(5.43)	8	(9.52)
Infectious disease	8	(2.99)	6	(3.26)	2	(2.38)
Vascular surgery	4	(1.49)	3	(1.63)	1	(1.19)
Gastro colorectal ward	3	(1.12)	0	(0.00)	3	(3.57)
Geriatrics	1	(0.37)	1	(0.54)	0	(0.00)
Gynaecology	12	(4.48)	8	(4.35)	4	(4.76)
Thyroid department	2	(0.75)	1	(0.54)	1	(1.19)
Cardiology department	3	(1.12)	2	(1.09)	1	(1.19)
Others	35	(13.06)	19	(10.33)	16	(19.05)
Diabetes						
No	228	(85.07)	153	(83.15)	75	(89.29)
Yes	40	(14.93)	31	(16.85)	9	(10.71)
Chronic obstructive pulmonary disease						
No	265	(98.88)	181	(98.37)	84	(100.00)
Yes	3	(1.12)	3	(1.63)	0	(0.00)
Hypertension						
No	202	(75.37)	137	(74.46)	65	(77.38)
Yes	66	(24.63)	47	(25.54)	19	(22.62)
Hepatopathy						
No	238	(88.81)	170	(92.39)	68	(80.95)
Yes	30	(11.19)	14	(7.61)	16	(19.05)
Tumor						
No	242	(90.30)	175	(95.11)	67	(79.76)
Yes	26	(9.70)	9	(4.89)	17	(20.24)
Artificial airway						
No	206	(76.87)	171	(92.93)	35	(41.67)
Yes	62	(23.13)	13	(7.07)	49	(58.33)
APACHE Ⅱ score						
Median(Q1,Q3)	21	(16,28)	20	(16,24)	27	(19,36)
Method of payment						
Basic medical insurance for urban employees	32	(11.94)	21	(11.41)	11	(13.10)
Basic medical insurance for urban and rural residents	70	(26.12)	44	(23.91)	26	(30.95)
Social assistance	1	(0.37)	0	(0.00)	1	(1.19)
Full public expense	4	(1.49)	4	(2.17)	0	(0.00)
Fullscale motor test sef cost	10	(3.73)	6	(3.26)	4	(4.76)
Other social insurance	2	(0.75)	2	(1.09)	0	(0.00)
Others	149	(55.60)	107	(58.15)	42	(50.00)
Allergic history						
No	253	(94.40)	174	(94.57)	79	(94.05)
Yes	15	(5.60)	10	(5.43)	5	(5.95)

Smoking history						
No	200	(74.63)	141	(76.63)	59	(70.24)
Yes	68	(25.37)	43	(23.37)	25	(29.76)
Drinking history						
No	233	(86.94)	163	(88.59)	70	(83.33)
Yes	35	(13.06)	21	(11.41)	14	(16.67)
Number of NSAIDs						
0	258	(96.27)	177	(96.20)	81	(96.43)
1	10	(3.73)	7	(3.80)	3	(3.57)
Number of anticoagulants						
0	173	(64.55)	112	(60.87)	61	(72.62)
1	95	(35.45)	72	(39.13)	23	(27.38)
Number of glucocorticoid						
0	110	(41.04)	77	(41.85)	33	(39.29)
1	147	(54.85)	99	(53.80)	48	(57.14)
2	11	(4.10)	8	(4.35)	3	(3.57)
3	-		-		-	

The data are presented as numbers(proportions) or the median(interquartile range).Median(Q1,Q3):Median(Quartile1,Quartile3).

STable 3 Univariate and multivariate logistic regression analysis of prevention group

Characteristics	Unadjusted OR（95% CI）	p-value	Adjusted OR （95% CI）	p-value
Gender				
Male	1.000（Reference）			
Female	1.524（0.999-2.325）	0.051		
Age（years）				
18-40	1.000（Reference）			
41-65	1.762（0.976-3.181）	0.060		
≥66	0.900（0.497-1.631）	0.728		
Transferred to department				
ICU	1.000（Reference）	0.002	1.000（Reference）	0.023
Orthopedics	0.000（0.000- ）	0.999	0.000（0.000- ）	0.999
Hepatobiliary surgery	22.533（4.648-109.247）	＜0.001	17.314(3.446-86.988)	0.001
Military sursery	4.815（0.953-24.322）	0.057	3.833(0.735-19.985)	0.111
Cardiothoracic surgery	4.333（0.544-34.544）	0.166	5.055(0.606-42.160)	0.134
Obstetrical department	3.824（1.001-14.612）	0.050	3.131(0.802-12.218)	0.100
Infectious disease	4.761（1.354-16.738）	0.015	4.360(1.209-15.719)	0.024
Vascular surgery	11.556（3.127-42.700）	＜0.001	9.577(2.513-36.498)	0.001
Gastro colorectal ward	13.481（3.133-58.008）	＜0.001	9.155(2.061-40.666)	0.004
Geriatrics	3.714（0.611-22.579）	0.154	3.661(0.581-23.079)	0.167
Gynaecology	3.852（0.719-20.624）	0.115	2.739(0.494-15.194)	0.249
Thyroid department	3.467（0.456-26.372）	0.230	4.171(0.525-33.121)	0.177
Cardiology department	8.667（1.671-44.939）	0.010	5.916(1.106-31.649)	0.038
Others	11.030（2.633-46.204）	＜0.001	9.412(2.144-41.318)	0.003

Diabetes				
No	1.000（Reference）			
Yes	0.551（0.301-1.007）	0.053		
Chronic obstructive pulmonary disease				
No	1.000（Reference）			
Yes	0.503（0.052-4.884）	0.554		
Hypertension				
No	1.000（Reference）			
Yes	1.335（0.847-2.104）	0.213		
Hepatopathy				
No	1.000（Reference）			
Yes	0.944（0.555-1.607）	0.833		
Tumor				
No	1.000（Reference）			
Yes	1.669（0.985-2.829）	0.057		
Artificial airway				
No	1.000（Reference）			
Yes	1.684（1.104-2.568）	0.016	1.455(0.896-2.364)	0.130
APACHE II score				
	0.995（0.975-1.016）	0.633		
Method of payment				
Basic medical insurance for urban employees	1.000（Reference）			
Basic medical insurance for urban and rural residents	1.604（0.823-3.124）	0.165		
Full public expense	0.000（0.000- ）	0.999		
Fullscale motor test sef cost	1.750（0.628-4.728）	0.270		
Other social insurance	2.1000（0.125-35.319）	0.606		
Others	1.430（0.781-23617）	0.246		
Allergic history				
No	1.000（Reference）			
Yes	2.114（0.868-5.147）	0.099		
Smoking history				
No	1.000（Reference）			
Yes	0.992（0.607-1.620）	0.974		
Drinking history				
No	1.000（Reference）			
Yes	0.898（0.500-1.611）	0.717		
Number of NSAIDs				
0	1.000（Reference）			
1	0.295（0.064-1.364）	0.118		
Number of anticoagulants				
0	1.000（Reference）			
1	0.430（0.266-0.696）	＜0.001	0.491(0.288-0.840)	0.009
Number of glucocorticoid				
0	1.000（Reference）	0.053	1.000（Reference）	0.701

1	1.048（0.647-1.697）	0.850	1.019(0.601-1.728)	0.943
2	0.353（0.147-0.847）	0.020	0.597(0.229-1.559)	0.292
3	0.353（0.038-3.277）	0.359	1.029(0.097-10.960)	0.981

Bold values indicate a p-value < 0.05.OR, odds ratio; CI, confidence interval.

STable 4 Univariate and multivariate logistic regression analysis of non-prevention group

Characteristics	Unadjusted OR（95% CI）	p-value	Adjusted OR（95% CI）	p-value
Gender				
Male	1.000（Reference）			
Female	1.439（0.853-2.427）	0.172		
Age（years）				
18-40	1.000（Reference）	0.164		
41-65	0.790（0.380-1.639）	0.526		
≥66	1.381（0.674-2.828）	0.377		
Transferred to department				
ICU	1.000（Reference）	0.022	1.000（Reference）	0.311
Orthopedics	0.623（0.220-1.770）	0.375	0.168(0.040-0.700)	0.014
Hepatobiliary surgery	0.063（0.014-0.277）	＜0.001	0.040(0.006-0.280)	0.001
Military sursery	0.216（0.043-1.091）	0.064	0.389(0.045-3.381)	0.392
Cardiothoracic surgery	0.172（0.020-1.522）	0.114	0.057(0.004-0.903)	0.042
Obstetrical department	0.216（0.057-0.814）	0.024	0.464(0.081-2.655)	0.388
Infectious disease	0.517（0.080-3.325）	0.487	0.087(0.010-0.746)	0.026
Vascular surgery	0.517（0.044-6.019）	0.599	0.317(0.012-8.225)	0.489
Gastro colorectal ward	0.000（0.000- ）	0.999	0.000（0.000- ）	0.999
Geriatrics	＞100（0.000- ）	1.000	＞100（0.000- ）	1.000
Gynaecology	0.345（0.075-1.593）	0.173	0.620(0.078-4.943)	0.652
Thyroid department	0.172（0.009-3.228）	0.240	0.176(0.003-11.518)	0.415
Cardiology department	0.345（0.026-4.557）	0.419	0.270(0.008-8.675)	0.460
Others	0.205（0.064-0.652）	0.007	0.103(0.021-0.500)	0.005
Diabetes				
No	1.000（Reference）			
Yes	1.688（0.765-3.727）	0.195		
Chronic obstructive pulmonary disease				
No	1.000（Reference）			
Yes	＞100（0.000- ）	0.999		
Hypertension				
No	1.000（Reference）			
Yes	1.174（0.638-2.158）	0.606		
Hepatopathy				
No	1.000（Reference）			
Yes	0.350（0.162-0.756）	0.008	0.752(0.250-2.265)	0.613
Tumor				
No	1.000（Reference）			

Yes	0.203（0.086-0.477）	＜0.001	0.335(0.100-1.136)	0.079
Artificial airway				
No	1.000（Reference）			
Yes	0.054（0.027-0.111）	＜0.001	0.037(0.011-0.123)	＜0.001
APACHE II score				
	0.919(0.891-0.949)	＜0.001	0.990(0.944-1.038)	0.675
Method of payment				
Basic medical insurance for urban employees	1.000（Reference）	0.899		
Basic medical insurance for urban and rural residents	0.886（0.369-2.218）	0.787		
Social assistance	0.000（0.000- ）	1.000		
Full public expense	＞100（0.000- ）	0.999		
Fullscale motor test sef cost	0.786（0.182-3.385）	0.746		
Other social insurance	＞100（0.000- ）	0.999		
Others	1.334（0.592-3.006）	0.486		
Allergic history				
No	1.000（Reference）			
Yes	0.908（0.300-2.744）	0.864		
Smoking history				
No	1.000（Reference）			
Yes	0.720（0.403-1.284）	0.266		
Drinking history				
No	1.000（Reference）			
Yes	0.644（0.310-1.339）	0.239		
Number of NSAIDs				
0	1.000（Reference）			
1	1.068（0.269-4.235）	0.926		
Number of anticoagulants				
0	1.000（Reference）			
1	1.705（0.970-2.996）	0.064		
Number of glucocorticoid				
0	1.000（Reference）	0.863		
1	0.884（0.518-1.508）	0.651		
2	1.143（0.285-4.580）	0.850		
3	-			

Bold values indicate a p-value < 0.05.OR, odds ratio; CI, confidence interval.
